# How does hard-to-reach status affect antiretroviral therapy adherence in the HIV-infected population? Results from a meta-analysis of observational studies

**DOI:** 10.1186/s12889-019-7135-0

**Published:** 2019-06-20

**Authors:** Dan Lin, Chun-yang Zhang, Zi-kai He, Xiao-dong Zhao

**Affiliations:** Fujian Center for Disease Control and Prevention / Fujian Provincial Key Laboratory of Zoonosis Research, 76 Jintai Road, Fuzhou, China

**Keywords:** Antiretroviral therapy, Adherence, HIV, Meta-analysis, Hard-to-reach population

## Abstract

**Background:**

Socially disadvantaged groups, such as drug users, sex workers and homeless individuals, are labelled as “hard-to-reach” (HTR) in public health and medical research. HIV disproportionately impacts these populations, but data on how the HTR status could affect antiretroviral therapy (ART) adherence among HIV-positive people are limited and have not been previously synthesized in a systematic manner. We performed a meta-analysis to explore the association between HTR status and optimal antiretroviral therapy adherence in the HIV-infected population to provide evidence and recommendations regarding ART adherence improvement and HIV infection control and prevention among HTR people.

**Methods:**

The PubMed, EMBASE, and Cochrance Library databases and the bibliographies of relevant studies were systematically searched up to December 2018. Full-text studies published in English were included, and no geographic or race restrictions were applied. Studies that quantitatively assessed the association between HTR status and optimal ART adherence among HIV-infected populations with a status of homelessness, sex work, or drug use were eligible for inclusion. We estimated the pooled odds ratios (ORs) of HTR characteristics related to ART adherence from each eligible study using a random effects model. The sensitivity, heterogeneity and publication bias were assessed.

**Results:**

Our search identified 593 articles, of which 29 studies were eligible and included in this meta-analysis. The studies were carried out between 1993 and 2017 and reported between 1999 and 2018. The results showed that HTR status resulted in a 45% reduction in the odds of achieving optimal ART adherence compared to odds in the general population (OR = 0.55, 95% confidential intervals (CIs) 0.49–0.62), and this significant inverse association was consistently found regardless of study design, exposure measurement, adherence cut-off points, etc. Subgroup analyses revealed that the HTRs tend to be suboptimal adhering during a longer observational period.

**Conclusions:**

HIV treatment adherence is extremely negatively affected by HTR status. It is crucial to develop appropriate interventions to improve ART adherence and health outcomes among HTR people who are HIV-infected.

**Electronic supplementary material:**

The online version of this article (10.1186/s12889-019-7135-0) contains supplementary material, which is available to authorized users.

## Background

Hard-to-reach (HTR) is a term used to describe those subgroups of the population who are difficult to reach or interact with [1] due to their behaviours, identities, or characteristics that lead to stigmatization and discrimination [2]. They generally consist of sex workers, drug users and homeless individuals [3] who are invisible in our daily life. The circumstances of their extremely disadvantaged state [4] cause them to be shunned by the public and the professionals who are in charge of providing them with support. These individuals who experience marked social exclusion are less likely than others to access healthcare services, and many of them face severe health inequities [5]. As human immunodeficiency virus (HIV) disproportionately impacts populations that suffer from health disparities, these marginalized people often face an increased risk of HIV infection compared to that of the general population. Findings [6–11] suggest that the overall prevalence rates of HIV infection among homeless people (1.24 to 1.7%), sex workers (8 to 17.3%) and drug users (17.7 to 34%) are relatively high. The estimated HIV prevalence in adults aged 15–49 years worldwide at the end of 2017 was 0.8% [12]. This implies that HIV infection is concentrated in these hidden groups whose behaviour exposes them to particularly high risks of acquiring or passing on HIV.

There is no known cure for HIV infection. However, effective antiretroviral drugs can control the virus and help prevent transmission so that people with HIV and those at substantial risk of acquiring it can enjoy long, healthy and productive lives [13]. The standard antiretroviral therapy (ART) maximally suppresses the HIV virus and stops the progression of HIV infection. Evidence has been available for several years that ART stops morbidity and mortality in HIV+ people and has clear benefits with regard to preventing the progression of HIV infection [14]. ART adherence plays a critical role in the treatment of HIV infection, and poor or suboptimal adherence has been associated with HIV treatment failure, with an insufficient viral suppression, a poor CD4 response, and an increased risk of developing drug resistance [15].

HTR populations, especially those who face multiple barriers to care, cycle in and out of optimal adherence of ART and thus cannot reap the life-prolonging benefits associated with strict adherence to the therapy. They may achieve superior adherence for a time and then drop out again when competing needs arise or their life circumstances change, leading to health-related challenges and threats among the group and even in the general public [16, 17]. In past few years, there has been a series of HIV outbreaks among this socially excluded group. The investigation into one HIV outbreak revealed that 157 cases were ultimately linked to only one infected drug user [18]. When another dramatic increase (1600%) in reported HIV-1 infections among injection drug users was noted, the largest transmission network included half of the analysed case samples, suggesting a limited number of sources and high levels of transmission networking among drug users [19]. Drug users may also serve as a bridge to the general population in the HIV epidemic [20]. HIV infection among heterosexual individuals with no history of injecting drugs was associated with having sexual partnerships with injection drug users [21]. A substantial proportion of male injection drug users are sex workers [20]. Sex workers, who exchange sex for material goods including drugs or money, have been shown to be a potential core group involved in for HIV transmission because they may facilitate the spread of HIV among their sexual networks. On the other hand, homelessness is an independent risk factor for HIV infection, and a lack of housing has been related to HIV outbreaks [22]. Homelessness is also strongly associated with the risky behaviours, including substance abuse, sexual intercourse without a condom, transactional sex, and multiple sexual partners. Such factors may facilitate risky sexual mixing patterns that promote the transmission of HIV.

The rate of adherence to highly active ART (HAART) by homeless people living with HIV ranges from 51% [23] to 89% [24]. Among HIV-infected female sex workers in low- and middle-income countries, the ART adherence is 76% [25]. Among HIV-infected drug users, the overall adherence is 60% [26]. Clinically, as a matter of fact, patients must take at least 95% of the prescribed antiretroviral doses in order to control viral replication [26] and achieve complete viral suppression. Unfortunately, it is difficult to achieve optimal adherence among these marginalized populations [27].

High rates of HIV prevalence and suboptimal adherence to ART are now seen among HTR populations, fostering the growing HIV epidemic. Because planning interventions to address suboptimal adherence to ART to control sources of infection is a critical approach to reduce HIV transmission among socially excluded HIV-infected individuals, there is growing interest in conducting research among HTR populations. Previous systematic reviews and/or meta-analyses have analysed the pooled rates of adherence to ART in a single HTR population. However, given the highly overlapping nature of these marginalised populations, with their common intersecting properties and adverse life experiences leading to similarly high levels of social exclusion, there is a lack of knowledge of how the HTR status may influence ART adherence. Elucidating how the negative properties shared by the HTR populations may affect optimal ART adherence is important to further our understanding of how to slow HIV transmission in these populations with high prevalence rates of HIV infection. We therefore examined how the HTR status (homelessness, engaging in sex work and drug use) could affect ART adherence among HIV-positive people and identified the gap in adherence between the socially included group and the HTR populations who experience considerable social exclusion and healthcare inequalities.

## Methods

### Search strategy

We searched for studies containing ART adherence outcomes in HTR populations (drug users, homeless individuals, and sex workers). In accordance with the Preferred Reporting Items for Systematic Reviews and Meta-Analyses (PRISMA) [28] guidelines, we developed a systematic literature search in electronic databases (PubMed, Cochrane Library and EMBASE) with no starting time limits, extending up to December 31, 2018, to identify studies that reported quantitative results regarding ART adherence by HIV-positive HTR individuals. The studies in the populations of interest had outcomes of effect sizes expressed as odds ratios (ORs). The search strategy was initially developed for the PubMed database and then adapted to the others. The following themes and keywords were then used to search for articles: (1) HIV infection: human immunodeficiency viruses OR HIV OR AIDS, (2) Antiretroviral therapy: ART OR HAART, (3) Adherence: adherence OR nonadherence OR suboptimal OR compliance OR noncompliance, and (4) HTR population: drug users OR sex workers OR homelessness. All four themes were then combined using the Boolean operator AND. References of eligible studies were hand-searched to identify additional relevant papers.

### Eligibility criteria

**Fig. 1:**
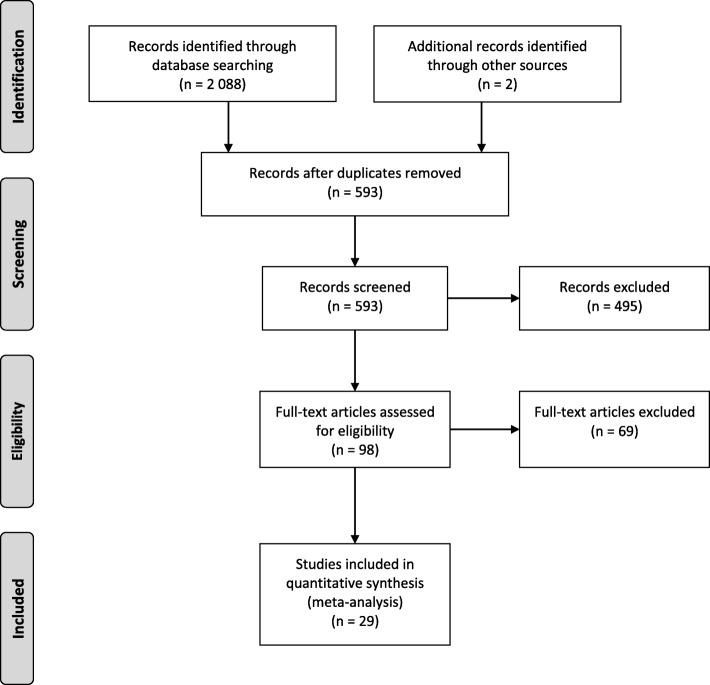
PRISMA flow diagram for the meta-analysis

Articles that met the following criteria were included in this meta-analysis: (1) the study quantitatively examined the effect size of the association between HTR status and ART adherence; (2) the study reported the adherence level (e.g., ≥ 95, 100%) or provided information that could be used to estimate the adherence; (3) the study applied a clear definition of the measurements and timeframe (e.g., self-reported, prescription refill, 1 week, or 1 month); and (4) the study was a peer-reviewed full-text articles published in English. Papers were not excluded on the basis of study design, sample size, exposure or outcome measure method, or geographical region [29], but they were excluded if (1) they were literature reviews, meta-analyses, laboratory studies, descriptive studies, or case reports, (2) the outcome presented was not ART adherence, (3) they were general-population-based studies, or (4) there was no effect size reported. If data were duplicated, we included the study with the longest observation period. The study selection process is detailed in Fig. 1.

### Data abstraction

We developed a standardized data collection form in accordance with the search criteria, and two reviewers (DL and CZ) independently performed the data extraction and comparison. All data were checked by a third reviewer (ZH) to systematically establish a dataset. Any discrepancies in the process were resolved by discussion with another investigator (XZ) or through reference to the original articles. The extracted items included the title, first author, publication year, geographic region, study design, number of participants, cut-offs values for adherence, adherence measures, survey period, measures of correlation (i.e., adjusted ORs), and summary descriptions of the study population. Duplicate data in different publications were excluded, and the study with the most informative and complete data was selected.

### Outcome measures

The outcomes were expressed as ORs in the eligible studies. The ORs could be directly used if the original study presented the association between any HTR population (homeless individuals, sex workers or drug users) and optimal ART adherence (optimal adherence to ART is often defined by a certain threshold, e.g., ≥ 95%, and this threshold differs across studies). To ensure maximum comparability of the outcomes across studies, we inverted the reported ORs if studies presented the association between suboptimal/nonadherence of ART and any characteristics of the socially excluded populations.

### Assessment of study quality

Two investigators (DL and CZ) independently assessed the quality of each study using the 9-star Newcastle-Ottawa Scale (NOS) [30] for cohort studies. The overall score ranges between 0 and 9 stars, and we considered a study awarded 7 or more stars to be a high-quality study. The methodological quality of the cross-sectional studies included was assessed using the 11-item checklist recommended by the Agency for Healthcare Research and Quality (AHRQ) [31] from the National Institutes of Health, and we considered a study awarded 8 or more stars to be a high-quality study [32]. Any disagreements were resolved through consensus.

### Data synthesis and statistical analyses

For the quantitative findings analysed in this study, outcomes expressed as ORs and their 95% confidential intervals (CIs) were extracted from each study. Crude (unadjusted) ORs or adjusted ORs were used directly in the pooled meta-analysis calculations. When ORs were generated from both univariable and multivariable models, the effect size from the multivariable model was used. All results were summarized in forest plots that showed the individual OR estimates.

Heterogeneity was tested using both the Q test and the I^2^ statistic. Cochran’s Q test was applied to qualitatively assess the heterogeneity across studies [33]. The Q test assesses whether differences in study estimates are due to chance alone (typically a *P* value< 0.1 or < 0.05 indicates heterogeneity among study estimates). The I^2^ statistic was used to quantify the extent of the heterogeneity and determine the proportion of the total variation in study estimates accounted for by it [34]. The results ranged from 0 to 100%, and a larger I^2^ indicates that the total variation between studies is due to true heterogeneity rather than sampling error (chance). The interpretation of the amount of heterogeneity is as follows [35]: I^2^ from 0 to 40%: might not be important; I^2^ from 30 to 60%: may represent moderate heterogeneity; I^2^ from 50 to 90%: may represent substantial heterogeneity; I^2^ from 75 to 100%: considerable heterogeneity. A Q-statistic value of *P* < 0.05 also suggests the presence of heterogeneity [9]. As we anticipated high levels of heterogeneity, a DerSimonian & Laird random effects model [36] was employed to pool the ORs across studies regardless of the significance of the between-study heterogeneity. Additionally, potential sources of heterogeneity were explored by stratifying the analyses by subgroups. We examined the robustness of the pooled effect estimates in defined subgroups (according to study design, study quality, adherence measures, etc.). Similarly, within-study heterogeneity was explored using random effects meta-regression analyses. Factors associated with effect sizes (pooled ORs) were investigated, and the results are reported as ORs with 95% CIs.

We examined the possibility of publication bias by generating funnel plots and performing meta-bias analyses (Begg’s test and Egger’s test). The publication bias for each outcome was accessed by evaluating asymmetry in the funnel plot, and ln(OR) was plotted against its standard error. Due to the subjective nature of graphical evaluation, Begg’s rank correlation test was also used to examine the asymmetry of the funnel plot [37]. Egger’s regression asymmetry test [38] was used to examine the association between the effect estimate and its variance. If an asymmetric funnel plot was identified, a contour-enhanced funnel plot was then used to further explore the source of bias [39]. All analyses were 2 tailed, with a *P* value < 0.05 indicating statistical significance. To evaluate the stability of the conclusions and the influence of individual studies, a one-study removed approach [40] was applied by omitting one study at a time to explore whether the pooled estimates were strongly influenced by any single study. All analyses were conducted with Stata version 15 for Windows (Stata Corporation, College Station, TX, USA).

## Results

### Literature search results

The electronic database search and manual search returned 2088 potentially relevant articles, and 1495 were excluded after title/abstract screening, resulting in 593 records for full text screening. Finally, 29 [41–69] studies that fulfilled the study entry criteria were included in our meta-analysis. The overall search flow is presented in Fig. 1.

### Study characteristics

Overall, 16 cohort studies and 13 cross-sectional studies were included in this meta-analysis. These studies were performed between 1993 and 2017 and reported between 1999 and 2018; the studies were conducted in several countries or regions. Most of the studies were from the United States (*n* = 15, 52%) and Canada (*n* = 7, 24%), and most of them investigated the association between drug use and ART adherence (*n* = 26, 90%). The measurement period for ART adherence ranged from 2 days to 1 year, and most used 100% (*n* = 13, 45%) or ≥ 95% (*n* = 10, 34%) as the thresholds for optimal adherence. Most studies measured adherence using self-reported questionnaires (*n* = 21, 72%), 7 studies used pharmacy refills, and only one study used the self-reporting plus pill count method. The characteristics of the eligible studies are summarized in Table 1.

**Table 1:** Characteristics of studies included in this meta-analysis

Author	Publication year	Study period	Region/Country	Study design	Population description	HTR status	Adherence threshold (%)	Adherence measure	Observation period	Quality assessment
Aloisi MS [41]	2002	1997–1999	Italy	Cohort	HIV-infected subjects who had never received antiretroviral therapy	DU	100	SR	6 months	Moderate
Avery AK [42]	2013	2008–2011	USA	Cross- sectional	Participants who were previously diagnosed HIV+	HL	90	SR	1 week	High
Biello KB [43]	2016	2012	Latin America	Cross- sectional	Men who have sex with men who reported being paid for sex with another man in the past year	SW	100	SR	1 month	Moderate
Bijker R [44]	2017	2007–2013	Asia	Cohort	HIV-infected adults starting first-line ART	DU	95	SR	6 months	High
Braitstein P [45]	2006	1996–2000	Canada	Cohort	Individuals used triple combination ART as their first HIV therapy and had documented HCV serology	DU	95	PR	1 year	High
Cohn SE [46]	2008	2002–2005	USA	Cohort	HIV-infected women between 20 and 34 weeks’ gestation	DU	100	SR	4 days or 3 months	Moderate
Cohn SE [47]	2011	1997–1999	USA	Cross- sectional	HIV-infected subjects without prior *Mycobacterium avium* complex (MAC) and with documented immune reconstitution	DU	100	SR	2 days	High
de Boni RB [48]	2016	2012–2013	Argentina, Brazil, Chile, Honduras, Mexico, and Peru	Cross- sectional	Individuals receiving care at HIV clinics	DU	100	SR	1 week	High
de Jong BC [49]	2005	2001	USA	Cross- sectional	Patients within a public health care system for HIV/AIDS	DU	100	SR	4 weeks	High
Duff PK [50]	2017	2014–2017	Canada	Cohort	Women living with HIV	DU	95	SR	3 to 4 weeks	High
Gebo KA [51]	2003	1999–2000	USA	Cross- sectional	Consecutive HIV-infected patients taking at least 1 antiretroviral medication	DU	90	SR	2 weeks	Moderate
Gordillo V [52]	1999	1997–1998	Spain	Cross- sectional	HIV-infected patients who were on treatment with antiretroviral drugs	DU	90	SR and pill count	1 week	High
Hicks PL [53]	2007	2003	USA	Cross- sectional	HIV patients on HAART in primary care	DU	95	SR	2 weeks	Moderate
Jin H [54]	2018	2013–2017	USA	Cross- sectional	HIV-positive men who have sex with men who had biologically confirmed, recent methamphetamine use	HL	90	SR	1 month	Moderate
Johnson MO [55]	2003	2000–2002	USA	Cross- sectional	HIV-positive adults taking ART	DU, HL	90	SR	3 days	Moderate
Joseph B [56]	2015	1996–2012	Canada	Cohort	HIV-infected people who use illicit drugs in a setting of universal no-cost HIV/AIDS treatment	HL, DU, SW	95	PR	6 months	High
King RM [57]	2012	2007–2009	USA	Cross- sectional	Persons living with HIV/AIDS who smoke	DU	100	SR	4 days	Moderate
Krusi A [58]	2010	1996–2007	Canada	Cohort	HIV-positive injection drug users	DU	95	PR	6 months	High
Mohanned H [59]	2004	1999–2001	USA	Cohort	Convenience sample of HIV-infected subjects seen at outpatient HIV clinics	DU	100	SR	1 week	High
O’Neil CR [60]	2012	2007–2010	Canada	Cohort	HIV-positive persons who have accessed antiretroviral therapy	DU	95	PR	1 year	High
Palepu A [61]	2004	1997–2002	Canada	Cohort	Participants who were HIV-infected, naive to HAART and who were prescribed treatment	DU	100	PR	6 months	High
Roux P [62]	2011	2006–2008	France	Cohort	HIV/HCV coinfected patients on ART	DU	100	SR	4 days	Moderate
Shannon K [63]	2005	2002–2004	Canada	Cohort	HIV-infected persons	DU	95	PR	6 months	Moderate
Sharpe TT [64]	2004	1997–2000	USA	Cross- sectional	HIV-infected women	DU	100	SR	not reported	Moderate
Stone VE [65]	2001	1993–1995	USA	Cross- sectional	Women living with HIV	DU	100	SR	3 days	Moderate
Teixeira C [66]	2013	2006–2008	Brazil	Cohort	HIV-positive patients with no previous ART	DU	95	SR	1 month	High
Tucker JS [67]	2003	1997–1998	USA	Cohort	People with known HIV infection and made at least one patient care visit	DU	100	SR	1 week	High
Turner BJ [68]	2000	1993–1997	USA	Cohort	Postpartum HIV-infected women	DU	80	PR	> 2 months	High
Wilson TE [69]	2002	1998–1999	USA	Cohort	HIV-positive women	DU	95	SR	6 months	Moderate

Abbreviations: *HTR* hard-to-reach, *DU* drug users, *HL* homelessness, *SW* sex workers, *SR* self-reporting, *PR* pharmacy refills

Of the 29 studies on ART adherence, 24 studies only investigated the association between drug use and ART adherence, 2 studies only studied the association between homelessness and ART adherence, and 1 study only reported the association between transaction sex and ART adherence. One study presented the relationships between ART adherence and all three HTR population characteristics, and one study presented the relationship between ART adherence and two HTR population characteristics (drug use and homelessness).

### Study quality

The quality assessments of the studies included in the analysis are listed in Additional file 1: Tables S1 and Table S2. The quality of the cohort studies ranged from 5 to 9 stars and was, on average, high, with a mean of 7 stars according to the NOS, while the quality of the cross-sectional studies ranged from 5 to 9 points and was, on average, moderate, with a mean AHRQ score of 7. The 16 cohort studies all described the selection of the non-exposed group, demonstrated that the outcome of interest was not present at the start of study, and followed the subjects long enough for the outcomes to occur. As the adherence measures for many cohorts were subjective (self-reported questionnaires), they had inaccurate outcome assessment and inadequate follow-up, leading to high levels of performance bias. However, performance bias due to confounding was low because of the reported adjustments. All 13 cross-sectional studies defined the source of information and indicated if the evaluators of the subjective components of study were blinded to other aspects of the status of the participants. Most of them, moreover, listed inclusion and exclusion criteria for exposed and unexposed subjects or referred to criteria used in previous publications, indicated whether the subjects were consecutive, and described the assessment and/or control of confounding variables. Similar to the cohort studies, many of the cross-sectionals did not describe undertaking any assessments for quality assurance purposes but clarified the expected follow-up and the percentage of patients for whom incomplete data or follow-up was obtained [30].

### HTR status and ART adherence

**Fig. 2:**
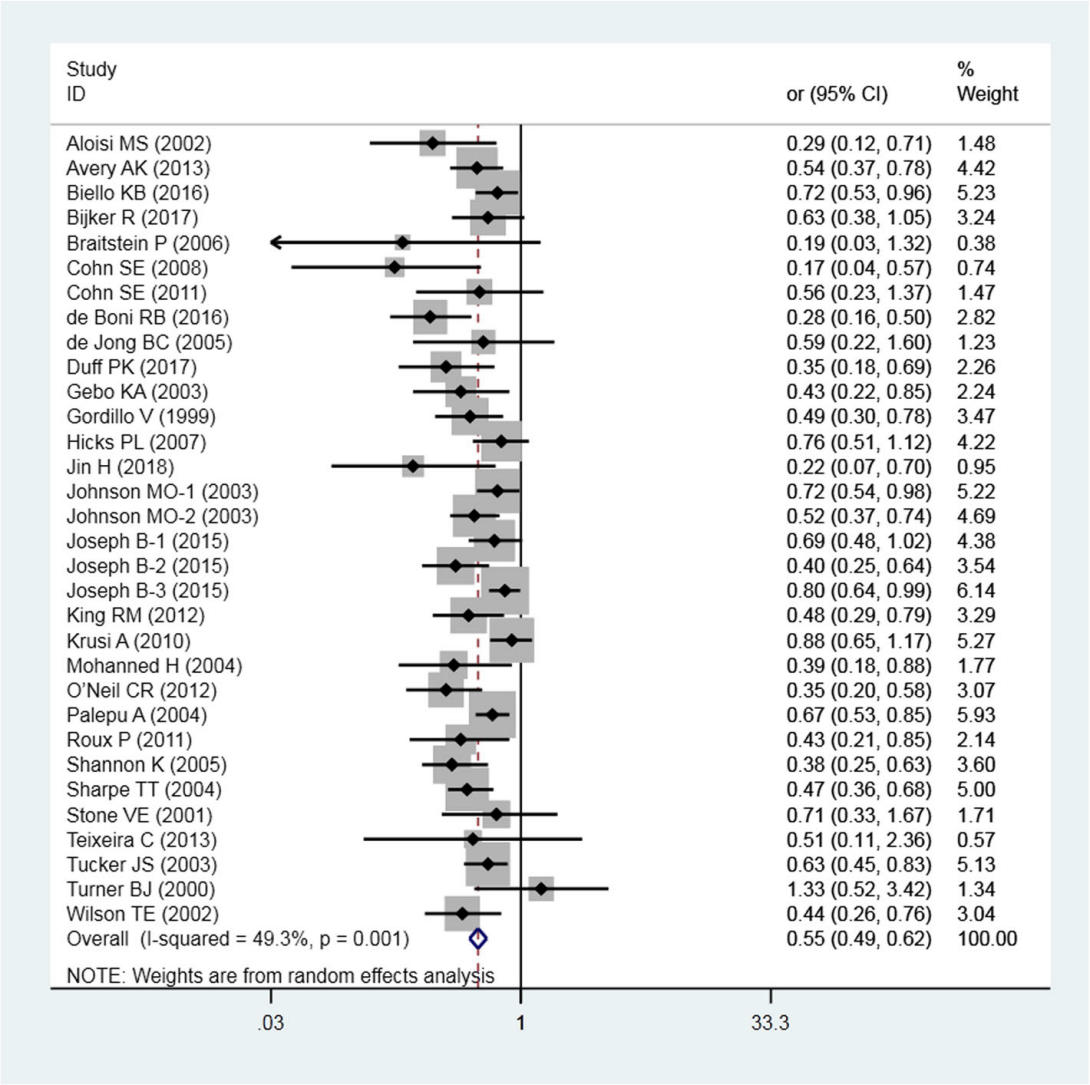
Pooled odds ratio for optimal ART adherence by HTR populations

Twenty-nine studies examined the impacts of HTR status on optimal ART adherence. The pooled association measures (ORs) and 95% CIs and those from individual studies are presented in Fig. 2. Despite individual study results varying widely from OR = 0.17 (95% CI 0.04–0.57) to OR = 1.33 (95% CI 0.52–3.42), the overall pooled estimate indicates that compared with the general population, HTR populations had 45% lower odds of achieving optimal ART adherence (OR = 0.55, 95% CI 0.49–0.62, *P* = 0.000). The I^2^ value was 49.3% (Q = 61.11, *P* = 0.001), indicating moderate statistical heterogeneity among the studies.

### Publication bias

We examined publication bias by plotting the log-transformed association measures (ORs) against their standard errors. Asymmetry was observed according to the visual inspection of the funnel plot, indicating the presence of publication bias (Additional file 2: Figure S1). A contour-enhanced funnel plot was used to aid in interpretation (Additional file 2: Figure S2) and demonstrated that the majority of studies had very low statistical significance. Hence, publication bias was a more likely cause of the funnel plot asymmetry. Clear publication bias was also detected out by Egger’s linear regression test (*P* = 0.000) (Additional file 2: Figure S3) and Begg’s test (*P* = 0.009). The trim-and-fill method [70], used to correct for publication bias, demonstrated that no studies needed to be filled.

### Sensitivity analyses

Leave-one study-out sensitivity analyses were conducted. The pooled estimate for optimal ART adherence ranged from OR = 0.57 (95% CI 0.53–0.62; when the study by Joseph [56] was excluded) to OR = 0.61 (95% CI 0.56–0.66; when the study by Sharpe [64] was excluded), suggesting that no study had undue influence on the pooled adherence estimate (Additional file 2: Figure S4).

### Meta-regression and subgroup analyses

We explored differences among studies and generated adjusted estimates by meta-regression and subgroup analysis as appropriate. The meta-regression covariates considered included study design, adherence threshold/cut-off point, adherence measure, region/country, observational period, and quality assessment results. Restricted maximum likelihood (REML) was applied to establish the regression model of OR-covariate. A *P* value of 0.03 was found for observational period, implying that the observational period was a source of between-study variance. When observational period was introduced into the regression model, the tau^2^ reduced from 0.05 to 0.04 and could explain 22.09% of the between-study variance. The proportion of within-study variance explained by the observational period was 24.14%. From the value of I-squared_res in the output, 42.47% of the residual variation was due to heterogeneity, with the other 57.53% attributable to within-study sampling variability.

We conducted subgroup analyses to recalculate the pooled ORs according to study design, adherence threshold/cut-off point, adherence measure, region/country, observational period, and quality assessment results (Table 2). Significant inverse associations between HTR status and ART adherence were observed in all subgroups, and all I^2^ test results indicated the presence of moderate heterogeneity within each subgroup [all *P* values (Q statistic) < 0.05]. In the interaction tests, the pooled ORs of the relationship between HTR and ART adherence in the subgroup with an observational period ≥6 months was 28% lower than that in the subgroup with an observational period < 6 months (OR value and its 95%CI for interaction: 0.72, 0.55–0.94, *P* value for interaction = 0.02), which meant that the HTR population is more likely to have suboptimal adherence during a longer observational period. However, there was no significant heterogeneity found in other subgroups.

**Table 2:** Odds ratios describing the association between HTR status and optimal ART adherence, categorized by subgroups

Subgroup	Number of measures of correlation	OR	95% CI		Tests for heterogeneity		Tests for interaction			
					*P* Value (Q Statistic)	I^2^ (%)	OR	95% CI		*P* value
Study design										
Cohort	18	0.54	0.45	0.65	0.00	58.3	0.98	0.77	1.25	0.88
Cross-sectional	14	0.55	0.47	0.64	0.00	31.4				
Adherence threshold/cut-off point (%)										
100	13	0.56	0.46	0.67	0.00	52.1	–			
≥ 95	12	0.52	0.42	0.66	0.00	54.1				
< 95	7	0.55	0.43	0.71	0.00	47.9				
Adherence measure										
Self-reporting	22	0.56	0.49	0.64	0.00	42.2	–			
Pharmacy refill	9	0.47	0.35	0.64	0.00	64.1				
Self-reporting & pill count	1	0.72	0.53	0.97	0.03	–				
Region/country										
USA	16	0.58	0.50	0.68	0.00	38.5	–			
Canada	9	0.44	0.34	0.58	0.00	51.7				
Others	7	0.60	0.47	0.78	0.00	58.5				
Observational period										
≥ 6 months	11	0.44	0.35	0.56	0.00	44.3	0.72	0.55	0.94	0.02
< 6 months	21	0.61	0.53	0.69	0.00	41.5				
Quality assessment results										
High	18	0.57	0.48	0.67	0.00	53.2	1.1	0.85	1.42	0.48
Moderate	14	0.52	0.44	0.65	0.00	41.3				

The number of studies was 29, but the number of measures of correlation was 32

## Discussion

### Main findings

There was extremely suboptimal adherence to ART in the HTR populations. We found a ratio of the pooled odds of optimal ART adherence in the HTR group to that in the general population of 0.55 (95% CI 0.49–0.62, *P* = 0.000), which means that compared with the general population, HTR populations have 45% lower odds of achieving optimal ART adherence. The relationships were markedly consistent across all subgroups (Table 2) and did not change in the sensitivity analyses (Additional file 2: Figure S4), adding further support for this conclusion.

We found a negative impact of social exclusion on adherence to HIV treatment, linking socioeconomic status to ART adherence. To the best of our knowledge, this is the first meta-analysis to comprehensively summarize the dire situation regarding suboptimal adherence to ART in socially excluded HTR populations. This meta-analysis reveals the gaps in ART adherence between HIV-infected people in the general population and those in HTR populations. In the context of the current focus on how combinations of social characteristics affect health [71, 72], we offer evidence of an association between health inequities and health outcomes. Health inequities are invisible social barriers created by ignorance and prejudice, and this discrimination hampers the equity of health services with regard to HIV treatment and transmission control. Therefore, policy makers and healthcare providers are the targets audience who need to be informed of the pooled data extracted from the existing studies involving health issues in socially excluded groups. These extreme inequities demand an intensive cross-sectoral policy and service response to prevent exclusion and improve health outcomes [73].

A meta-analysis of adherence to HAART by HIV+ patients in China revealed that the adherence over 1 month and ≥ 3 months was 80.9 and 68.3%, respectively, indicating an inverse association between adherence and treatment duration [74]. Likewise, we obtained similar results in the subgroup analysis, which indicated that the pooled effect size in the subgroup with an observational period ≥6 months was significantly lower than that in the subgroup with an observational period < 6 months. The gap (28%) between the ORs confirmed the suboptimal adherence to HIV treatment. However, data on ART adherence from HTR individuals with an observational period ≥6 months is scarce (9 studies with an observational period ≥6 months VS 20 studies with an observational period < than 6 months), although those studies reported the lowest pooled OR (OR = 0.44, 95%CI: 0.35–0.56, *P* = 0.000) across all subgroups in this meta-analysis. Due to the relative lack of data, the adherence to ART by HTR individuals for longer than 6 months should be addressed as a matter of priority in future research.

The reasons for lower adherence to ART in the HTR population are complicated. For drug users, it is unlikely that a single mechanism explains the adverse impact of active substance use on adherence. The results from a cohort study implied that the use of stimulants (i.e., cocaine or methamphetamine) proved to be particularly disruptive to adherence to therapy by HIV-infected adults, and adherence was most dramatically affected during periods of active stimulant use. There are also several potential mechanisms by which substance use may impact adherence behaviour, including neurocognitive deficits, psychosocial impairment, and exacerbation of psychiatric dysfunction [75]. Nevertheless, others have suggested that poor adherence among substance users in general may be due to their inconsistent, unpredictable, and chaotic lifestyles [76]. In addition, as demonstrated in other studies, in the settings with free ART access for sex workers, an array of individual, social and structural barriers to poor adherence were identified, such as stigma and discrimination in healthcare settings, a lack of support, the criminalisation of sex work for HIV-infected individuals, and geographic mobility [77, 78]. On-going drug use and poverty may also indirectly prevent sex workers from achieving optimal ART adherence. Furthermore, a study conducted by Royal SW et al. investigated adherence to HAART by homeless persons living with HIV/AIDS [19]. The results showed that coexisting problems of limited access to healthcare, an elevated risk of mental health problems, and worse attitudes toward treatment are associated with an increased likelihood of worse adherence. Others argued that barriers to care, including the lack of financial resources, lack of transportation, side effects, restrictions on when and how the medications should be taken and stored, and insufficient health insurance coverage, may be especially problematic for homeless individuals [79].

### Limitations

The interpretation of the findings should be considered in the context of the limitations of this study. We grouped different types of HTR populations (drug users, sex workers and homeless individuals) and believe that there is commonality with regard to their HIV treatment adherence owing to overlap in their experience of marked social exclusion. But we were unable to identify a sufficient number of studies performed with homeless and sex worker populations (only 2 studies included for sex workers and 4 included homeless individuals) and believe that further work is needed to specifically describe their HIV treatment experiences. And the representativeness of our analysis is limited for three reasons. First, publication bias was generated by our collection of papers published in English. As with most meta-analyses, the information reported in this source is restricted. Second, there was uniformity of the geographical distribution of the included studies. Fifteen studies were from the USA, and 7 studies were from Canada, resulting in an unequal weighting of the pooled estimates and making it impossible to generalize our findings to HTR populations in specific contexts or in a generally resource-poor settings. For example, in Eastern Europe, HIV infection is mainly driven by drug use, and access to HAART is uneven [80]. Last but not least, although we confirmed that the subjects investigated in Canada were from different research programs, most of the studies were based in the same city (Vancouver), which suggests the possibility of duplicated data and overly narrow CIs. Another limitation is the reporting bias that can be introduced by the methods used for measuring adherence. Twenty-one out of the 29 studies included in our analysis measured ART adherence by self-report questionnaires. Although self-reporting is the standard method of collecting behavioural information, it is not as accurate as objective measurements, such as electronic devices and concentrations of the medication in the blood [74], leading to overestimation of ART adherence. Furthermore, a considerable proportion of the observed heterogeneity may be explained by differences [81] in adherence thresholds, observational periods and study designs. The cut-off value for ART adherence ranged from 80 to 100%, and the recall time frame ranged from 2 days to 1 year. The absence of universal definitions of cut-off points and recall time frames for the measurement of ART adherence is likely to explain some of the variation. To use the most informative and complete data in cases of data duplication or overlap, we selected the study with the longest observation time. However, unsurprisingly, and consistent with findings from longitudinal studies of adherence [82], the adherence rates clearly decreased over time, and the participants dropped out, which introduced selection bias into our study. Meanwhile, all studies included in our meta-analyses were observational in design; randomized controlled trial data were not available. In addition, a random effects model was chosen based on the moderate level of heterogeneity [47] in the overall analysis, but substantial heterogeneity remained in the subgroup analysis, as is often the case in the meta-analysis of epidemiological studies [74]. Finally, although the included studies controlled for many important confounders, such as age, gender, race, education and employment status, it is still possible that there was residual confounding due to the presence of unknown confounders and/or imprecise adjustment strategies [29].

## Conclusion

Adherence to ART is a key predictor of survival for HIV-infected people. HTR populations have endured a high burden of HIV infection during and between HIV epidemics. Suboptimal adherence to ART by HTR populations could be associated with clinical failure, the emergence of viral resistance and, subsequently, the potential for on-going HIV transmissions and outbreaks. The combined evidence from this meta-analysis indicated that HTR populations have suboptimal adherence to ART. The data were consistent across different subgroups, suggesting that being socially excluded is a potential risk factor for severe outcomes in people living with HIV. Our findings regarding ART adherence by HTR people who suffer from extreme health inequities have implications for public health and medical service provision. Developing strategies and policies to address these inequities is essential for providing sustainable assistance and support.

## Additional files

Additional file 1: **Table S1.** Quality assessment of included cohort studies. **Table S2.** Quality assessment of included cross-sectional studies. (DOCX 30 kb)

Additional file 2: **Figure S1.** Funnel plot used to explore the source of publication bias. **Figure S2.** Contour-enhanced funnel plot used to explore the source of publication bias. **Figure S3.** Egger’s linear regression test used to explore the source of publication bias. **Figure S4.** Sensitivity analyses for assessing the impact of individual studies on the pooled estimate. (DOCX 5749 kb)

## Data Availability

All data generated or analysed in this study are included in this article and its additional files.

## Abbreviations

AHRQ: Agency for Healthcare Research and Quality
ART: Antiretroviral therapy
CI: Confidential interval
DU: Drug users
HAART: Highly active ART
HIV: Human immunodeficiency virus
HL: Homelessness
HTR: Hard-to-reach
MAC: *Mycobacterium avium* complex
NOS: Newcastle-Ottawa Scale
OR: Odds ratios
PR: Pharmacy refills
PRISMA: Preferred Reporting Items for Systematic Reviews and Meta-Analyses
REML: Restricted maximum likelihood
SR: Self-reporting
SW: Sex workers
