# Supplementary material for: How does hard-to-reach status affect antiretroviral therapy adherence in the HIV-infected population? Results from a meta-analysis of observational studies
Source: BMC Public Health. 2019 Jun 20;19:789. doi: 10.1186/s12889-019-7135-0 (PMC6587270; doi:10.1186/s12889-019-7135-0)
Supplement: Supplementary file 1 — Table S1. Quality assessment of included cohort studies. Table S2. Quality assessment of included cross-sectional studies. (DOCX 30 kb) [file 12889_2019_7135_MOESM1_ESM.docx]

**Table S1** Quality assessment of included cohort studies

| Study | Year | Selection | | | | Comparability | Outcome | | | Quality assessment | Grade |
| --- | --- | --- | --- | --- | --- | --- | --- | --- | --- | --- | --- |
|  |  | Representativeness of the exposed cohort | Selection of the non exposed cohort | Ascertainment of exposure | Demonstration that outcome of interest was not present at start of study | Comparability of cohorts on the basis of the design or analysis | Assessment of outcome | Was follow-up long enough for outcomes to occur | Adequacy of follow up of cohorts |  |  |
| Aloisi MS [41] | 2002 | 0 | 1 | 0 | 1 | 2 | 0 | 1 | 0 | 5 | moderate |
| Bijker R [44] | 2017 | 1 | 1 | 0 | 1 | 2 | 0 | 1 | 1 | 7 | high |
| Braitstein P [45] | 2006 | 1 | 1 | 1 | 1 | 2 | 1 | 1 | 1 | 9 | high |
| Cohn SE [46] | 2008 | 1 | 1 | 0 | 1 | 2 | 0 | 1 | 0 | 6 | moderate |
| Duff PK [50] | 2017 | 1 | 1 | 1 | 1 | 2 | 0 | 1 | 0 | 7 | high |
| Joseph B [56] | 2015 | 0 | 1 | 1 | 1 | 2 | 1 | 1 | 0 | 7 | high |
| Krusi A [58] | 2010 | 0 | 1 | 1 | 1 | 2 | 1 | 1 | 0 | 7 | high |
| Mohanned H [59] | 2004 | 1 | 1 | 1 | 1 | 2 | 0 | 1 | 1 | 8 | high |
| O’Neil CR [60] | 2012 | 1 | 1 | 1 | 1 | 2 | 1 | 1 | 0 | 8 | high |
| Palepu A [61] | 2004 | 1 | 1 | 1 | 1 | 2 | 1 | 1 | 1 | 9 | high |
| Roux P [62] | 2011 | 0 | 1 | 0 | 1 | 2 | 0 | 1 | 0 | 5 | moderate |
| Shannon K [63] | 2005 | 1 | 1 | 0 | 1 | 0 | 1 | 1 | 1 | 6 | moderate |
| Teixeira C [66] | 2013 | 1 | 1 | 1 | 1 | 2 | 0 | 1 | 0 | 7 | high |
| Tucker JS [67] | 2003 | 1 | 1 | 1 | 1 | 2 | 0 | 1 | 1 | 8 | high |
| Turner BJ [68] | 2000 | 1 | 1 | 1 | 1 | 2 | 1 | 1 | 0 | 8 | high |
| Wilson TE [69] | 2002 | 0 | 1 | 1 | 1 | 2 | 0 | 1 | 0 | 6 | moderate |

**Table S2** Quality assessment of included cross-sectional studies

| Study | Avery AK [42] | Biello KB [43] | Cohn SE [47] | de Boni RB [48] | de Jong BC [49] | Gebo KA [51] | Gordillo V [52] | Hicks PL [53] | Jin H [54] | Johnson MO [55] | King RM [57] | Sharpe TT [64] | Stone VE [65] |
| --- | --- | --- | --- | --- | --- | --- | --- | --- | --- | --- | --- | --- | --- |
| Year | 2013 | 2016 | 2011 | 2016 | 2005 | 2003 | 1999 | 2007 | 2018 | 2003 | 2012 | 2004 | 2001 |
| 1) Define the source of information (survey, record review) | 1 | 1 | 1 | 1 | 1 | 1 | 1 | 1 | 1 | 1 | 1 | 1 | 1 |
| 2) List inclusion and exclusion criteria for exposed and unexposed subjects (cases and controls) or refer to previous publications | 1 | 1 | 1 | 1 | 1 | 1 | 0 | 1 | 1 | 0 | 1 | 1 | 1 |
| 3) Indicate time period used for identifying patients | 1 | 1 | 1 | 1 | 1 | 1 | 1 | 1 | 1 | 0 | 1 | 1 | 1 |
| 4) Indicate whether or not subjects were consecutive if not population-based | 1 | 1 | 1 | 1 | 1 | 0 | 1 | 1 | 1 | 1 | 1 | 1 | 1 |
| 5) Indicate if evaluators of subjective components of study were masked to other aspects of the status of the participants | 1 | 1 | 1 | 1 | 1 | 1 | 1 | 1 | 1 | 1 | 1 | 1 | 1 |
| 6) Describe any assessments undertaken for quality assurance purposes (e.g., test/retest of primary outcome measurements) | 0 | 0 | 0 | 0 | 0 | 0 | 1 | 0 | 1 | 1 | 0 | 0 | 1 |
| 7) Explain any patient exclusions from analysis | 1 | 0 | 0 | 0 | 0 | 0 | 1 | 0 | 0 | 1 | 0 | 0 | 0 |
| 8) Describe how confounding was assessed and/or controlled. | 1 | 1 | 1 | 1 | 1 | 1 | 1 | 1 | 0 | 1 | 1 | 0 | 0 |
| 9) If applicable, explain how missing data were handled in the analysis | 1 | 0 | 0 | 1 | 1 | 0 | 0 | 0 | 0 | 1 | 0 | 1 | 0 |
| 10) Summarize patient response rates and completeness of data collection | 1 | 1 | 1 | 0 | 1 | 0 | 1 | 0 | 0 | 0 | 0 | 0 | 0 |
| 11) Clarify what follow-up, if any, was expected and the percentage of patients for which incomplete data or follow-up was obtained | 0 | 0 | 1 | 1 | 0 | 0 | 0 | 0 | 0 | 0 | 0 | 0 | 0 |
| Quality assessment | 9 | 7 | 8 | 8 | 8 | 5 | 8 | 6 | 6 | 7 | 6 | 6 | 6 |
| Grade | high | moderate | high | high | high | moderate | high | moderate | moderate | moderate | moderate | moderate | moderate |
