# Supplementary material for: How does hard-to-reach status affect antiretroviral therapy adherence in the HIV-infected population? Results from a meta-analysis of observational studies
Source: BMC Public Health. 2019 Jun 20;19:789. doi: 10.1186/s12889-019-7135-0 (PMC6587270; doi:10.1186/s12889-019-7135-0)
Supplement: Supplementary file 2 — Figure S1. Funnel plot used to explore the source of publication bias. Figure S2. Contour-enhanced funnel plot used to explore the source of publication bias. Figure S3. Egger’s linear regression test used to explore the source of publication bias. Figure S4. Sensitivity analyses for assessing the impact of individual studies on the pooled estimate. (DOCX 5749 kb) [file 12889_2019_7135_MOESM2_ESM.docx]

**Fig. S1.** Funnel plot used to explore the source of publication bias


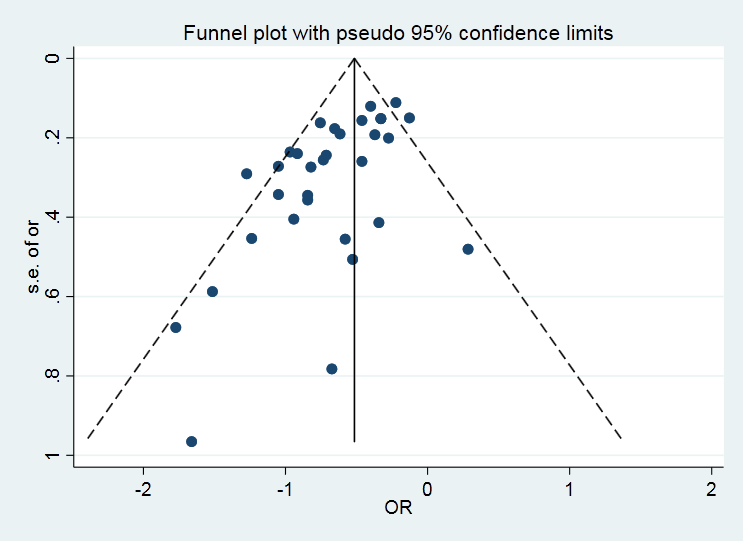


**Fig. S2.** Contour-enhanced funnel plot used to explore the source of publication bias


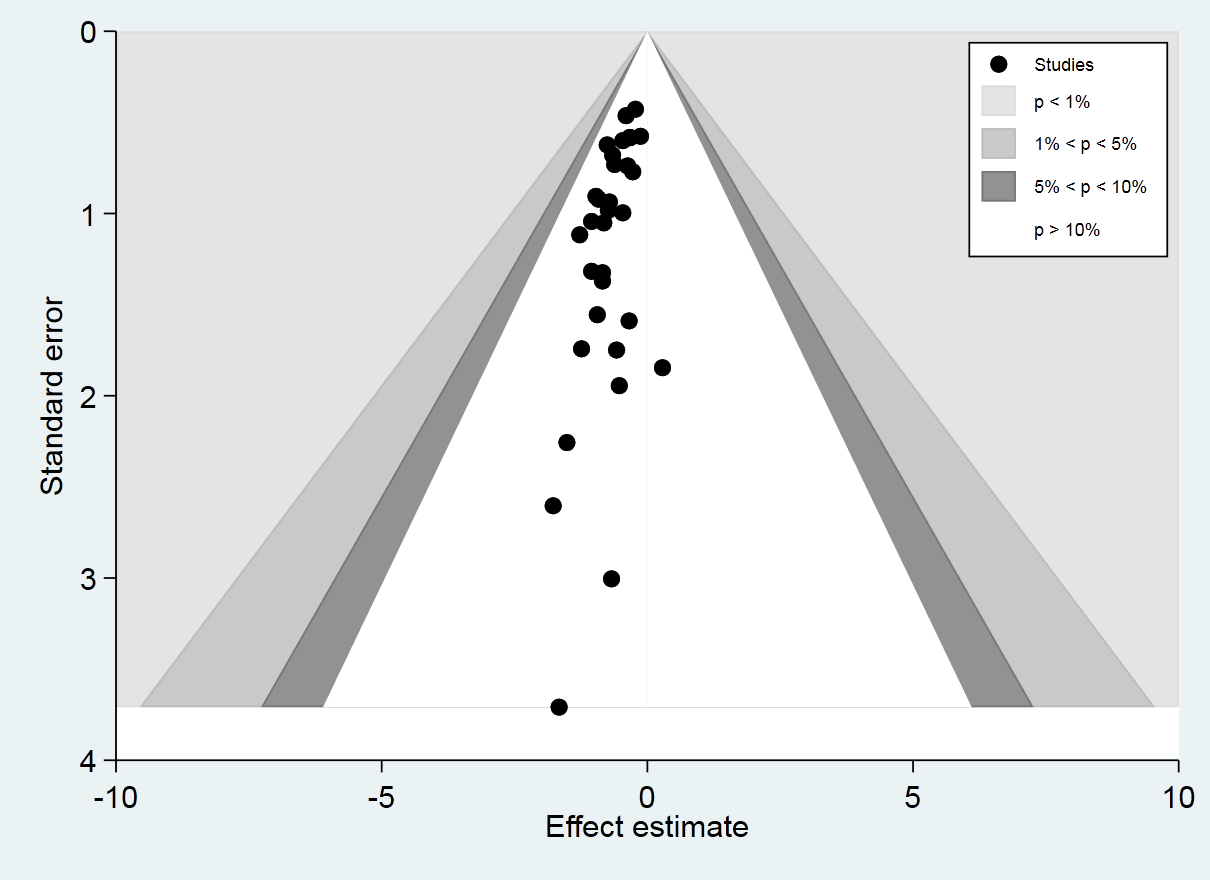


**Fig. S3.** Egger’s linear regression test used to explore the source of publication bias


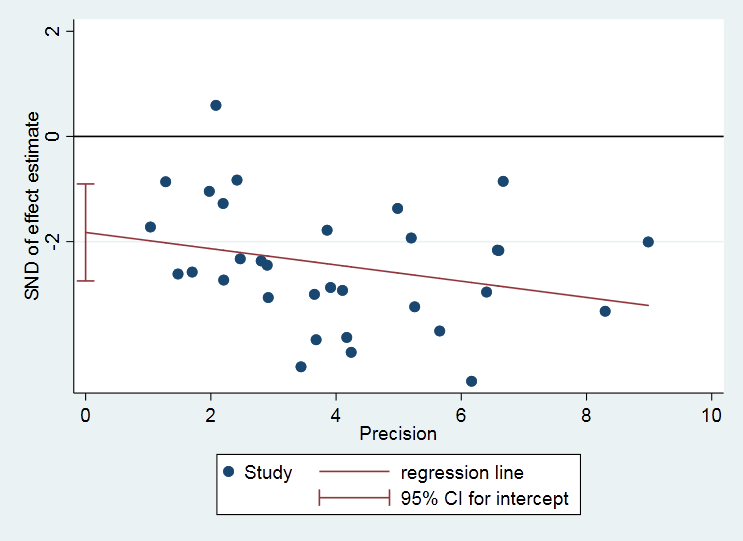

**Fig. S4.** Sensitivity analyses for assessing the impact of individual studies on the pooled estimate
